# Estimating antibiotic coverage from linked microbiological and clinical data from the Swiss Paediatric Sepsis Study to support empiric antibiotic regimen selection

**DOI:** 10.3389/fped.2023.1124165

**Published:** 2023-05-11

**Authors:** Aislinn Cook, Andrew Atkinson, Andreas Kronenberg, Philipp K. A. Agyeman, Luregn J. Schlapbach, Swiss Pediatric Sepsis Study Group, Christoph Aebi, Christoph Berger, Julia Anna Bielicki

**Affiliations:** ^1^ Centre for Neonatal and Paediatric Infection, St. George’s University of London, London, United Kingdom; ^2^ Health Economics Research Centre, Nuffield Department of Population Health, University of Oxford, Oxford, United Kingdom; ^3^ Pediatric Research Centre, University Children's Hospital Basel, Basel, Switzerland; ^4^ Institute for Infectious Diseases, University of Bern, Bern, Switzerland; ^5^ Department of Pediatrics, Inselspital, Bern University Hospital, University of Bern, Bern, Switzerland; ^6^ Department of Intensive Care and Neonatology, Children's Research Center, University children's Hospital Zürich, Zürich, Switzerland; ^7^ Division of Infectious Diseases and Hospital Epidemiology, Children’s Research Center, University Children’s Hospital Zurich, Zurich, Switzerland

**Keywords:** sepsis, paediatrics, antibiotic treatment, empiric antibiotic therapy, antibiotic resistance, coverage, WISCA

## Abstract

In light of rising antibiotic resistance, better methods for selection of empiric antibiotic treatment based on clinical and microbiological data are needed. Most guidelines target specific clinical infections, and variably adjust empiric antibiotic selection by certain patient characteristics. Coverage estimates reflect the probability that an antibiotic regimen will be active against the causative pathogen once confirmed and can provide an objective basis for empiric regimen selection. Coverage can be estimated for specific infections using a weighted incidence syndromic combination antibiograms (WISCAs) framework. However, no comprehensive data combining clinical and microbiological data for specific clinical syndromes are available in Switzerland. We therefore describe estimating coverage from semi-deterministically linked routine microbiological and cohort data of hospitalised children with sepsis. Coverage estimates were generated for each hospital and separately pooling data across ten contributing hospitals for five pre-defined patient risk groups. Data from 1,082 patients collected during the Swiss Paediatric Sepsis Study (SPSS) 2011–2015 were included. Preterm neonates were the most commonly represented group, and half of infants and children had a comorbidity. 67% of neonatal sepsis cases were hospital-acquired late-onset whereas in children 76% of infections were community-acquired. *Escherichia coli*, Coagulase-negative staphylococci (CoNS) and *Staphylococcus aureus* were the most common pathogens. At all hospitals, ceftazidime plus amikacin regimen had the lowest coverage, and coverage of amoxicillin plus gentamicin and meropenem were generally comparable. Coverage was improved when vancomycin was included in the regimen, reflecting uncertainty about the empirically targeted pathogen spectrum. Children with community-acquired infections had high coverage overall. It is feasible to estimate coverage of common empiric antibiotic regimens from linked data. Pooling data by patient risk groups with similar expected pathogen and susceptibility profiles may improve coverage estimate precision, supporting better differentiation of coverage between regimens. Identification of data sources, selection of regimens and consideration of pathogens to target for improved empiric coverage is important.

## Introduction

Early concordant therapy, defined as antibiotic treatment that is subsequently shown to be active *in vitro* against the identified causative bacteria, appears to be associated with improved survival for children with sepsis, in particular among vulnerable populations, such as neonates (1). One challenge for achieving concordance is that antibiotic treatment for severe infections, such as sepsis, is generally started empirically, when the causative pathogen is unknown. This presents a dilemma for clinicians and those setting hospital guidelines regarding recommendations for empiric regimens. Already, a shift towards the more intensive use of very broad-spectrum agents from the World Health Organization AWaRe classification (2–4) Watch group is being observed in regions with a high prevalence of resistant bacteria (5). In many low and middle-income countries, for example, meropenem is now the most commonly used empiric therapy for neonates and children treated for sepsis (5). While using broader spectrum regimens may improve concordance, using these antibiotics sparingly or only when necessary is important to reduce the development of resistance and conserve their utility.

Better methods are required to improve targeting of empiric regimen guidelines, for example by targeting specific infection syndromes or by incorporating knowledge of certain patient characteristics in the selection of regimens. It is likely that this would result in a reduction in use of broader-spectrum agents for patients unlikely to benefit from them, but a more targeted and consistent use of such antibiotics empirically in certain high-risk situations or patient groups. Where local microbiology data is used to inform empiric prescribing guidelines, it is commonly done using aggregate hospital antibiograms (e.g., all ages) or surveillance data without accounting for differing epidemiology by patient characteristics which may be problematic because of inherent issues of bias, small sample sizes and limited clinical relevance (6–9).

One approach that has been described and combines microbiological and clinical data is to calculate expected coverage of antibiotic regimens based on a weighted incidence syndromic combination antibiogram (WISCA) (10–12). Syndromic coverage estimates use routine microbiological data from patients with specific infections, for example children with bloodstream infections, to assess the probability that a specific regimen will cover episodes of that syndrome, improving clinical relevance. Clinical relevance might be further increased by accounting for specific patient characteristics (13) in coverage estimates.

Datasets combining microbiological surveillance and clinical information are not often routinely available. Basing coverage estimates on microbiological data from a single hospital alone, especially when focusing on isolates identified in a specific infection syndrome, considerably reduces sample sizes, which limits the utility of coverage estimates due to wide uncertainty intervals by now allowing for the differentiation between regimens (11). Borrowing or pooling data can be one way to deal with this challenge, especially if microbiological patterns are expected to be similar for specific patients across different hospitals.

The aim of this study was to generate a linked dataset combining surveillance microbiological data and clinical characteristics for neonates and children with blood-culture positive sepsis enrolled in a prospective cohort. These linked data were used to describe coverage of common empiric regimens in Swiss paediatric populations exploring either using data from a single hospital or pooling within patient groups across hospitals in Switzerland.

## Materials and Methods

We addressed the study's objectives by exploring and selecting appropriate methods of data linkage between the Swiss National Surveillance database ANRESIS and a prospectively collected cohort of neonates and children with blood culture positive sepsis. Based on the linked data, antibiotic coverage was then estimated for various antibiotic regimens in use for empiric treatment of paediatric sepsis, using different stratification approaches for data pooling.

### Datasets

#### Clinical data

The Swiss Paediatric Sepsis Study (SPSS) of neonates and children with blood-culture proven sepsis collected data between September 1, 2011 and December 31, 2015 at 10 participating hospitals in Switzerland (14). The primary aim of SPSS was to assess the epidemiology and to identify immune defects associated with severe sepsis and sepsis-mortality, and detailed demographic and epidemiological data related to the sepsis episode were collected including underlying comorbidities, hospitalisation prior to culture, empiric treatment and causative pathogen. Causative pathogens were recorded using a drop-down list of key species (see Supplementary Material) or by providing additional details in a free text field. Antimicrobial susceptibility testing data collected in SPSS were limited to key resistance phenotypes, such as methicillin-resistance in *Staphylococcus aureus* or extended-spectrum beta-lactamase expression in Gram-negative bacteria. These do not allow a full estimation of coverage of different antibiotic regimens in children with blood-culture proven sepsis in Switzerland, instead requiring linkage to microbiological data including full antimicrobial susceptibility testing results. Of note, pathogens that are frequently considered contaminants, such as coagulase-negative staphylococci, (CoNS) were considered relevant in SPSS if the participants fulfilled all study criteria for sepsis, the local investigator considered the identified pathogen causative, treatment was targeted towards this pathogen and given for at least 5 days.

#### Microbiological data

Detailed antimicrobial susceptibility data from centres participating in SPSS during the same time period were obtained from ANRESIS, a national bacteraemia and antibacterial resistance surveillance database collecting routine data provided by Swiss microbiology laboratories (15). Speciation and antimicrobial susceptibility testing data, for example, for blood culture isolates, including potential contaminants, are electronically extracted from participating laboratories' information management systems according to a predefined algorithm and entered into the surveillance database. Demographic data collected are limited to age and sex, but the source hospital, date of hospitalisation and date of blood culture are also available. Local laboratory sample numbers are recorded, enabling manual identification of relevant samples by the laboratory transferring data, but all patient identifiable data are anonymised within ANRESIS. Participation is voluntary in ANRESIS, but all centres contributing to SPSS were also represented in ANRESIS.

### Linkage methods

Both probabilistic and semi-deterministic linkage approaches were evaluated for quality of linking the SPSS study data to the detailed susceptibility data from ANRESIS. The process, validation and outcomes of both linkage approaches are described in the Supplementary Material.

As the semi-deterministic linkage approach had a higher proportion of cases linked and a lower rate of falsely linked cases, the semi-deterministically linked dataset was used for further analysis. Briefly, semi-deterministic linkage took a stepwise approach. We started out with matches on all data items available in both databases (site, age group, gender, pathogen and day of blood culture). As a next step we accepted a widened window for the date of blood culture, expecting matching on all other items. We then included some “fuzzy”-type matching rules for the pathogen field, described in the Supplementary Material. Finally, both age and blood culture date matching criteria were relaxed or omitted. For each of the above steps, where multiple records were matched, we chose the one with the nearest blood culture date, or failing this arbitrarily the first record. Those records not matched in any of the steps were identified and manually reviewed. This latter set was also an interesting source of finding new potential matches for the fuzzy matching process.

### Data definitions

#### Regimens of interest

The regimens of interest for the coverage calculation were: amoxicillin + gentamicin, amoxicillin/clavulanic acid, amoxicillin/clavulanic acid + gentamicin, cefepime, ceftriaxone, ceftazidime + amikacin, piperacillin/tazobactam and meropenem. In supplementary analyses, we also looked at coverage when adding vancomycin as part of the regimen.

#### Susceptibility interpretations for coverage estimates

After linkage, all patients with fungal species or organisms that were considered contaminants were removed from the dataset. Reported results in ANRESIS were coded as susceptible (S), intermediate (I) and resistant (R). Results reported as intermediate were classified as resistant for the purposes of this analysis. EUCAST interpretive algorithms were used to interpret the reported resistance results from ANRESIS for the selected antibiotics. Briefly,

• Where the organism is considered by EUCAST to be intrinsically resistant to the antibiotic of interest, it was coded as resistant regardless of reported susceptibility.
• Primarily, reported susceptibility to the antibiotic of interest was used.
• If susceptibility to the antibiotic of interest was not available, susceptibility was inferred from other antibiotics in the same class (e.g., susceptibility to gentamicin was inferred from other aminoglycosides if gentamicin susceptibility was not reported).
• Susceptibility to cephalosporins and carbapenems for *S. aureus* and Coagulase-negative Staphylococci was inferred from cefoxitin or oxacillin reported results, where available.
• For vancomycin, all streptococci were considered susceptible regardless of what was reported given EUCAST indication that resistant isolates are rare.

Susceptibility to a regimen comprised of more than one antibiotic was determined as the pathogen being classified susceptible to at least one antibiotic in the regimen; resistant was where it was resistant to all antibiotics in the regimen; unknown/not tested was where results were unavailable for both antibiotics or unavailable for one antibiotic when the other antibiotic in the regimen was reported resistant.

#### Patient risk groups

Using basic demographic and patient data collected as part of the SPSS data, we grouped patients into five risk groups based on a system previously used in antibiotic use surveillance (13). The groupings are based on basic risk factors assumed to be associated with different underlying epidemiology or empiric guidance. The groups are as follows:

• Group 1 – neonates with community-acquired sepsis
• Group 2 – neonates with hospital-acquired sepsis
• Group 3 – infants and children without comorbidities with community-acquired sepsis
• Group 4 – infants and children with comorbidities with community-acquired sepsis
• Group 5 – infants and children with hospital-acquired sepsis

Neonates were defined as term-born infant less than 28 days at blood culture sampling or prematurely-born infant less than 44 weeks gestational age at blood culture sampling. Community-acquired sepsis was defined as culture taken ≤2 days after admission and hospital-acquired sepsis was >2 days after admission. In neonates, community-acquired sepsis includes both early-onset sepsis (culture taken ≤2 days of life) and late-onset community-acquired sepsis (culture taken ≤2 days after admission in babies presenting from the community). In non-neonates, we defined comorbidities according to the paediatric complex chronic conditions classifications system version 2 (14, 16) We considered infants and children with comorbidities to be those with at least one comorbidity regardless of its type.

### Data analysis

Weighted-incidence syndromic combination antibiograms (WISCA) were used to estimate coverage to the chosen empiric regimens. As previously described (11, 12, 17), the WISCA is conceptualised as a Bayesian decision tree approach accounting for relative incidence of causative pathogens producing a point estimate of coverage as the mean of the posteriors and a 95% credible interval for each regimen of interest. We used non-informative priors for relative incidence and susceptibility. Incidence is assumed to come from a multinomial Dirichlet distribution (with K the numbers of each pathogen and alpha equal to 1) and susceptibility is assumed to come from a beta distribution (with the two shape parameters taken from the number of susceptible isolates and the number of non-susceptible isolates). We estimated the WISCAs using a Monte-Carlo approach with 1,000 simulations producing an average estimated coverage for the regimen of interest with associated 95% credible interval.

Coverage for regimens was calculated for each hospital using all isolates in each hospital and separately for patient groups by pooling isolates across hospitals for each patient group. Pathogens where all isolates had unknown susceptibility results for the regimen of interest were excluded for the coverage calculation of that regimen.

Data linkage, data cleaning and modelling for coverage analyses was conducted in R v4.1.2 (18).

### Ethical approval

The SPSS study was approved by the ethics committees of all participating centres (Cantonal Ethics Committee, Inselspital, University of Bern, no KEK-029/11) (14). ANRESIS is a surveillance database with fully anonymised data therefore no ethics approvals were required.

## Results

After removing those with no pathogen identified in ANRESIS, with a species considered a contaminant, or with a fungal pathogen, 1,082 patients from 10 hospitals participating in the Swiss Paediatric Sepsis Study with organism and susceptibility data matched from ANRESIS were included in the coverage analysis (Supplementary Figure S1).

The number of patients included per facility ranged between 49 and 224. All hospitals covered the whole paediatric age-spectrum, except for one which was exclusively a neonatal unit. Overall, the most frequently represented age group was preterm neonates (24%, *n* = 258) followed by children aged one to four years (20% *n* = 218) and children <1 year (20%, *n* = 215). 52% of children (>28 days of life) had a comorbidity (369/712); neonates were considered their own category of comorbidity given their age (Table 1). 67% of infections (248/370) in neonates were hospital-acquired late-onset sepsis whereas in children, 76% (541/712) of infections were community-acquired infections (CAI).

**Table 1:** Summary characteristics of the Swiss Paediatric Sepsis Study (SPSS) patients included in this analysis.

Characteristic	*N* = 1 082^a^
**Hospital**	
Hospital 1	56 (5%)
Hospital 2	89 (8%)
Hospital 3	164 (15%)
Hospital 4	57 (5%)
Hospital 5	80 (7%)
Hospital 6	174 (16%)
Hospital 7	83 (8%)
Hospital 8	49 (5%)
Hospital 9	106 (10%)
Hospital 10	224 (21%)
**Age group**	
Preterm neonate	258 (24%)
Term neonate	112 (10%)
Child <12 months	215 (20%)
Child 1–4 years	218 (20%)
Child 5–9 years	141 (13%)
Child 10–16 years	138 (13%)
**Sex**	
Female	435 (40%)
Male	647 (60%)
**Comorbidity**	
Neonate	370 (34%)
Comorbidity	369 (34%)
No Comorbidity	343 (32%)
**CAI/HAI^b^**	
Early-onset neonatal sepsis	62 (6%)
Late-onset sepsis CAI	60 (6%)
Late-onset sepsis HAI	248 (23%)
Community-acquired infection (CAI)	541 (50%)
Hospital-acquired infection (HAI)	171 (16%)
**Patient on PICU**	534 (49%)
**Derived risk group**	
Group 1 - neonatal sepsis CAI	122 (11%)
Group 2 - neonatal sepsis HAI	248 (23%)
Group 3 - CAI healthy children	328 (30%)
Group 4 - CAI comorbidity children	213 (20%)
Group 5 - HAI children	171 (16%)

^a^ Frequency (%).

^b^ CAI, community-acquired infection (≤2 days from hospital admission to blood culturing); HAI, hospital-acquired infection (>2 days from hospital admission to blood culturing).

After grouping patients into risk groups, 30% of episodes (*n* = 328) occurred in previously healthy children with community-acquired infections, and 23% in neonates with hospital-acquired infections (*n* = 248). The least common risk groups were neonates with community-acquired infections (11%, *n* = 122) and hospital-acquired infections (HAI) in children (16%, *n* = 171) (Table 1). Number of patients per risk group at each hospital are presented in Supplementary Table S1.

Overall, there were 34 unique pathogens identified in all patients. The most common pathogens were *Escherichia. coli* (21%, *n* = 231), Coagulase-negative Staphylococcus (CoNS) (17%, *n* = 178), *S. aureus* (16%, *n* = 172) and *Streptococcus pneumoniae* (11%, *n* = 116) (Supplementary Table S2 and Supplementary Figure S2, S3).

**Figure 1:**
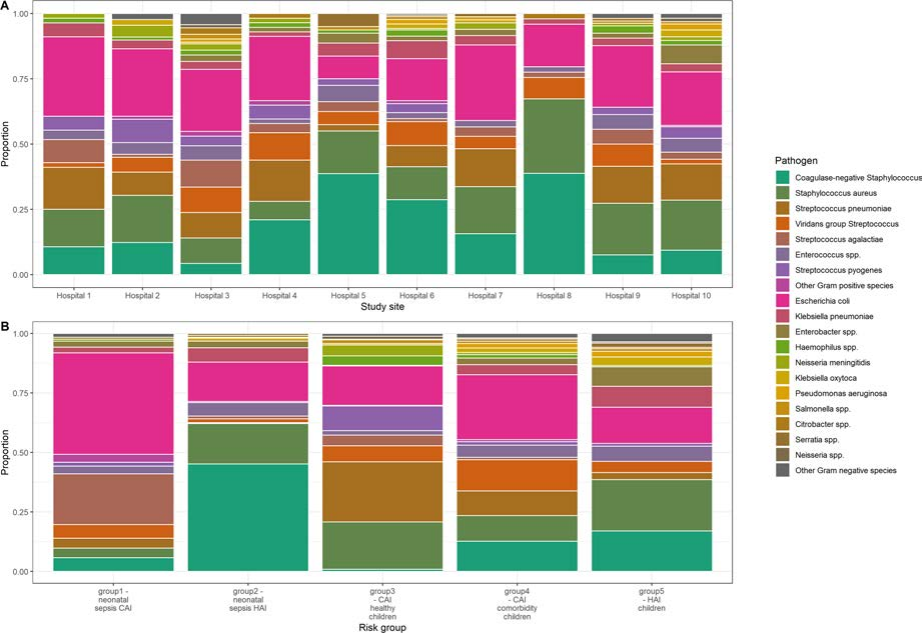
Distribution of pathogens included in the study by (**A**) hospital and (**B**) patient risk group.

By site, the most common pathogen was *E. coli* at 7 sites (range: 24%, 25/106–30%, 17/56) and CoNS at 3 sites (range: 29%, 50/174–39%, 19/49 and 31/80) (Figure 1A). By patient risk group, the most common pathogen in group 1 was *E. coli* (43%, 54/122), group 2 was CoNS (45%, 112/248), group 3 was *S. pneumoniae* (25%, 83/328), group 4 was *E. coli* (27%, 58/213) and group 5 was *S. aureus* (22%, 37/171) (Figure 1B).

### Coverage

**Figure 2:**
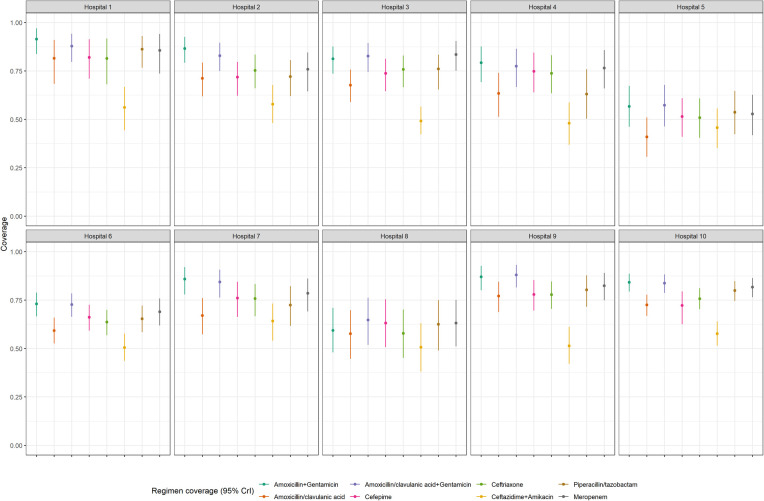
Coverage probability estimates and 95% credible intervals for empiric antibiotic regimens of interest at each hospital.

Full coverage results for each hospital are shown in Figure 2 and described in Supplementary Table S3 Considering coverage by site, the regimen with the lowest coverage at all hospitals was ceftazidime plus amikacin ranging from 48% to 64% with overlapping credible intervals. One site had noticeably lower coverage for all regimens compared to other hospitals, with data reflecting mostly episodes among neonates. Coverage of amoxicillin plus gentamicin ranged between 57% and 91%. Credible intervals were wide at most sites, reflecting the low number of isolates (<100 at 6/10 sites) contributing to the coverage models.

**Figure 3:**
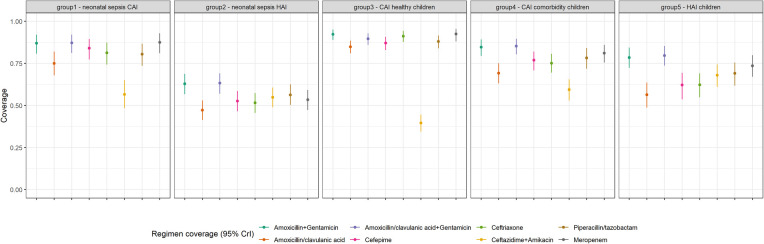
Coverage probability estimates and 95% credible intervals for empiric antibiotic regimens of interest for each patient risk group.

When looking at coverage by patient risk group (Figure 3 and Supplementary Table S4), coverage and precision were the highest in group 3 – healthy children with CAI for all regimens except ceftazidime plus amikacin and credible intervals were the smallest (most robust estimates). Group 2 – neonates with HAIs - had low coverage (<65%) for all regimens of interest. Patients with HAIs had significantly lower coverage to amoxicillin/clavulanic acid (non-overlapping credible intervals) but coverage was significantly improved with the addition of gentamicin.

**Figure 4:**
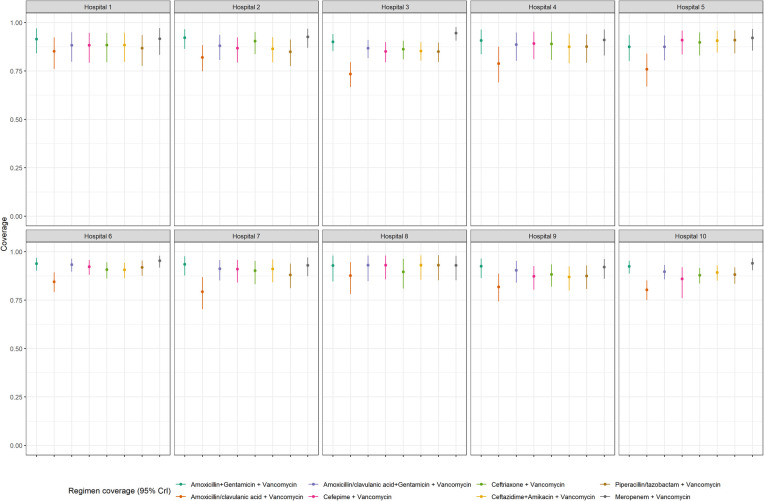
Coverage probability estimates and 95% credible intervals for empiric antibiotic regimens PLUS vancomycin at each hospital.

**Figure 5:**
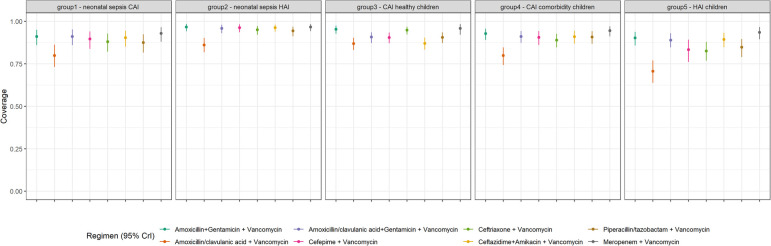
Coverage probability estimates and 95% credible intervals for empiric antibiotic regimens PLUS vancomycin for each patient risk group.

We performed a supplementary analysis adding vancomycin to all regimens of interest for coverage estimates at hospitals (Figure 4 and Supplementary Table S5) and for patient groups (Figure 5 and Supplementary Table S6). Across the board, coverage estimates increased, and credible intervals became smaller with the addition of vancomycin. The most notable coverage increases were seen at hospital 5 and in neonates with HAIs, most likely linked to the high proportion of blood culture positive sepsis episodes due to coagulase-negative staphylococci in both instances. Adding vancomycin to regimens for community-acquired infections in children without comorbidities (group 3), did not provide much of an improvement in coverage estimates although credible intervals did become smaller.

## Discussion

We linked detailed clinical data from a Swiss Paediatric Sepsis cohort study with a national microbiology surveillance dataset to support estimation of antibiotic coverage of commonly used empiric regimens. Semi-deterministic matching to link clinical characteristics with routine microbiology data was successful with >90% match success. We calculated coverage in two ways: (i) for each hospital, reflecting information that would be found in a paediatric-specific hospital antibiogram; (ii) for five pre-defined patient risk groups pooling data across hospitals, selecting patient risk groups that might be expected to have a similar microbiological epidemiology. We focused on typical regimens used in Switzerland as first choice empiric treatment for sepsis. Generally, these do not provide cover for methicillin-resistant *S. aureus* (MRSA), as MRSA prevalence in Switzerland is low (19).

At hospital-level, coverage of amoxicillin plus gentamicin (range 57%–91% of episodes covered) was highest or comparable to meropenem (53%–96%), whereas ceftazidime plus amikacin offered the lowest coverage. Because of limited sample size, differentiation of coverage between different regimens was limited in some sites.

Considering patient risk groups, coverage was lowest for all regimens in group 2 – neonates with HAI (range 47%–63%) and highest in group 3 – healthy children with CAIs (85%–93%). Ceftazidime plus amikacin performed worst, with an estimated coverage of only 40% of episodes in group 3, for example. Coverage of all antibiotic regimens except ceftazidime plus amikacin followed the same pattern when children in the risk groups 3–5 were considered: estimated coverage was highest for group 3 – healthy children with CAI, lower for group 4 – children with comorbidities, and lowest for children experiencing HAI. This is in line with studies in adult populations indicating that an important risk factor for lower coverage is whether infections are community-acquired, healthcare-associated or hospital acquired (20, 21). The decline in expected coverage in groups 3–5 was most pronounced for beta-lactams used alone without addition of an aminoglycoside.

In neonates with HAIs, coagulase-negative staphylococci were most commonly identified in blood culture as causing the sepsis episode (22), likely contributing to the low coverage of the regimens of interest given high reported methicillin-resistance of up to 90% (23, 24). Similarly, lower coverage of all regimens at two sites, one comprising only a neonatal unit and the other predominantly having recruited neonates, is likely to have been related to the high frequency of coagulase-negative staphylococci in this subgroup.

With a relevant proportion of patients in SPSS considered to have sepsis related to coagulase-negative staphylococci and enterococci, we included a supplemental analysis of coverage of empiric regimens adding vancomycin. At hospital level, the most substantial increase in estimated coverage to estimates without vancomycin was observed at sites predominantly enrolling neonates into SPSS. Similarly, the impact on coverage of adding vancomycin was most striking for patient risk group 2 – neonates with HAI,but was also considerable for the other two groups reflecting healthcare-associated infections: group 4 – children with comorbidities presenting from the community and group 5 – children with HAI. There was no improvement in coverage estimates in children without comorbidities with CAIs (group 3) when adding vancomycin potentially due to the lower number of CoNS identified in these infections.

There is on-going debate whether improved early coverage offered by adding vancomycin is associated with survival benefits (25), and therefore whether incorporating vancomycin in empiric regimens is appropriate (25, 26). The addition of vancomycin has been shown to be important in settings where MRSA contributes substantially to paediatric bloodstream infections, which is not the case in Switzerland (27, 28). Instead, the consensus is to add vancomycin for management of childhood sepsis or bloodstream infection in certain situations, for example in response to identification of coagulase-negative staphylococci in culture as targeted treatment, when indicated.

Our analysis points to a number of critical aspects of clinically relevant coverage estimates for informing empiric antibiotic regimen selection. First, it is essential to define the bacterial species that are considered critical. If there is no clinical advantage of early coverage of methicillin-resistant coagulase-negative staphylococci, for example, these isolates should not contribute to coverage estimates. With such an approach, coverage for the standalone beta-lactam and beta-lactam/aminoglycoside combinations for the remaining pathogens are expected to be high. This is important, because clinicians express a clear preference for regimens providing coverage of at least 80% and 90% for mild and severe sepsis, respectively (29), and may be driven to unjustified broad-spectrum agent combinations unless the species targeted by empiric therapy are clarified and reflected.

Second, any decisions on target bacteria for coverage estimates impact sample size, especially at hospital level. This in turn results in wider 95% credible intervals, differentiation between coverage point estimates becomes limited and selection of certain regimens over others consequently more difficult. Pooling data for patients considered to share a risk profile for the target infection responds to this problem. Our analysis supports this as an appropriate approach, in particular for CAI in any age group, where patterns of causative bacteria are highly comparable between sites. Healthcare-associated infections (group 4) and HAI are characterised by greater variability in causative pathogens, and potentially in antimicrobial resistance, being more reflective of what is happening locally. Detected or undetected local outbreaks or endemicity of certain strains could then considerably distort relative incidence and resistance prevalence, respectively (11). Estimating within-patient risk group coverage with data from several hospitals is likely to be acceptable in a low-resistance setting, such as Switzerland.

Third, there is on-going work to determine the best mode of presenting coverage estimates. In the US, a human-centred antibiogram has been developed providing the coverage shortfall of regimens based on patient risk factors and for specific infection syndromes (30). In a prototype, coverage estimates are based on all causative bacteria, without limiting input data to those for pathogens most important for early concordant treatment. This results in similar “coverage gaps” of typical broad-spectrum beta-lactam backbones as in our study, due to high prevalence of vancomycin-resistant enterococci and coagulase-negative staphylococci.

Our study has several limitations. We did not account for some patients contributing multiple isolates to the SPSS and ANRESIS datasets because of polymicrobial infections. This means that our coverage estimates reflect the distribution of bacterial isolates in both datasets and not necessarily the treated episodes. The linkage process itself may have introduced bias if matches between demographics and organisms were not a perfect match; however, we feel the linkage process was well evaluated and few patients were not matched. Furthermore, estimated coverage is for patients enrolled in the SPSS and may not be entirely reflective of neonates and children with blood-culture positive sepsis treated in Swiss hospitals more generally. Bias may have been introduced by differential lack of enrolment of patients with mild or severe sepsis, for example, as these may be expected to have a different microbiological epidemiology. Conversely, it is not possible to identify children with sepsis and bloodstream infection from ANRESIS directly, since relevant clinical data are not collected.

## Conclusion

Global and national guidance recommends using local epidemiology to inform empiric prescribing policies, however this can be difficult if sample sizes are small and patient groups are heterogenous within a hospital. Underlying incidence of causative pathogens and resistance may vary between types of patients at one facility (for example neonates vs. older children, healthy children vs. children with comorbidities, community vs. hospital-acquired infections). Therefore, empiric guidelines accounting for these basic characteristics may be more impactful and have greater uptake by clinicians. Pooling data in this way also results in more precise coverage estimates reliably differentiating better performing regimens. This requires expanding typical microbiological surveillance with some clinical information, most importantly relating to the infection that was being treated at the time of sampling. Where these data are not routinely collected together, we illustrate that linkage methods are feasible and provided robust matching (∼90%). For Switzerland, we observed that more conservative regimens in line with antibiotic stewardship tenets, such as amoxicillin plus gentamicin, are expected to perform in a comparable manner to more broad-spectrum options, including meropenem. Such similar coverage between narrower and broader regimens could encourage a local-data-driven rationale for continuing to use narrower regimens.

## Data Availability

The raw data supporting the conclusions of this article will be made available by the authors, without undue reservation.

## References

[1] Cook A Hsia Y Russell N Sharland M Cheung K Grimwood K Association of empiric antibiotic regimen discordance with 30-day mortality in neonatal and pediatric bloodstream infection — a global retrospective cohort study. J Pediatr Infect Dis. (2021) (2021) 40(2):137–43. doi: 10.1097/INF.0000000000002910 10.1097/INF.0000000000002910 33395208

[2] Brink AJ Mendelson M. Be AWaRe: new metrics for paediatric antibiotic stewardship. Lancet Infect Dis. (2019) 19(1):6–7. 10.1016/S1473-3099(18)30557-7 30522835

[3] Sharland M Cappello B Ombajo LA Bazira J Chitatanga R Chuki P The WHO AWaRe antibiotic book: providing guidance on optimal use and informing policy. Lancet Infect Dis. (2022) 22(11):1528–30. 10.1016/S1473-3099(22)00683-1 36309019

[4] Sharland M Pulcini C Harbarth S Zeng M Gandra S Mathur S Classifying antibiotics in the WHO essential medicines list for optimal use—be AWaRe. Lancet Infect Dis. (2018) 18(1):18–20. 10.1016/S1473-3099(17)30724-7 29303731

[5] Jackson C Hsia Y Basmaci R Bielicki J Heath PT Versporten A Global divergence from world health organization treatment guidelines for neonatal and pediatric sepsis. J Pediatr Infect Dis. (2019) 38(11):1104–6. 10.1097/INF.0000000000002433 31425329

[6] Ashley EA Dance DAB Turner P. Grading antimicrobial susceptibility data quality: room for improvement. Lancet Infect Dis. (2018) 18(6):603–4. 10.1016/S1473-3099(18)30273-1 29856355

[7] Lim C Hantrakun V Teerawattanasook N Srisamang P Teparrukkul P Sumpradit N Impact of low blood culture usage on rates of antimicrobial resistance. J Infect Dis. (2021) 82(3):355–62. 10.1016/j.jinf.2020.10.040 PMC7994019 33278401

[8] Rempel OR Laupland KB. Surveillance for antimicrobial resistant organisms: potential sources and magnitude of bias. Epidemiol Infect. (2009) 137(12):1665–73. 10.1017/S0950268809990100 19493372

[9] Turner P Fox-Lewis A Shrestha P Dance DAB Wangrangsimakul T Cusack TP Microbiology Investigation Criteria for Reporting Objectively (MICRO): a framework for the reporting and interpretation of clinical microbiology data. BMC Med. (2019) 17(70). 10.1186/s12916-019-1301-1 PMC6440102 30922309

[10] Hebert C Ridgway J Vekhter B Brown EC Weber SG Robicsek A. Demonstration of the weighted-incidence syndromic combination antibiogram: an empiric prescribing decision aid. Infect Control Hosp Epidemiol. (2012) 33(4):381–8. 10.1086/664768 22418634

[11] Bielicki JA Sharland M Johnson AP Henderson KL Cromwell DA Berger C Selecting appropriate empirical antibiotic regimens for paediatric bloodstream infections: application of a Bayesian decision model to local and pooled antimicrobial resistance surveillance data. J Antimicrob Chemother. (2016) 71(3):794–802. 10.1093/jac/dkv397 26626717

[12] Cook A Sharland M Yau Y Group PB Bielicki J. Improving empiric antibiotic prescribing in pediatric bloodstream infections: a potential application of weighted-incidence syndromic combination antibiograms (WISCA). Expert Rev Anti Infect Ther. (2022) 20(3):445–56. 10.1080/14787210.2021.1967145 34424116

[13] Bielicki JA Sharland M Versporten A Goossens H Cromwell DA. Using risk adjustment to improve the interpretation of global inpatient pediatric antibiotic prescribing. PLoS One. (2018) 13(7):e0199878. 10.1371/journal.pone.0199878 29979795 PMC6034826

[14] Agyeman PKA Schlapbach LJ Giannoni E Stocker M Posfay-Barbe KM Heininger U Epidemiology of blood culture-proven bacterial sepsis in children in Switzerland: a population-based cohort study. Lancet Child Adolesc Health. (2017) 1(2):124–33. 10.1016/S2352-4642(17)30010-X 30169202

[15] Methods - ANRESIS. Available at: https://www.anresis.ch/antibiotic-resistance/methods/ (Cited Dec 9, 2022).

[16] Feudtner C Feinstein JA Zhong W Hall M Dai D. Pediatric complex chronic conditions classification system version 2: updated for ICD-10 and complex medical technology dependence and transplantation. BMC Pediatr. (2014) 14(1). 10.1186/1471-2431-14-199 PMC4134331 25102958

[17] Bielicki JA Sharland M Heath PT Walker AS Agarwal R Turner P Evaluation of the coverage of 3 antibiotic regimens for neonatal sepsis in the hospital setting across Asian countries. JAMA Netw Open. (2020) 3(2):e1921124. 10.1001/jamanetworkopen.2019.21124 32049298 PMC11893050

[18] R Core Team. R: A Language and Environment for Statistical Computing. Vienna, Austria: R Foundation for Statistical Computing (2022). Available at: https://www.R-project.org/

[19] Olearo F Albrich WC Vernaz N Harbarth S Kronenberg A. Staphylococcus aureus and methicillin resistance in Switzerland: regional differences and trends from 2004 to 2014. Swiss Med Wkly. (2016) 146(37):w14339. 10.4414/smw.2016.14339. 27631162

[20] Shorr AF Tabak YP Killian AD Gupta V Liu LZ Kollef MH. Healthcare-associated bloodstream infection: a distinct entity? Insights from a large U.S. Database. Crit Care Med. (2006) 34(10):2588–95. 10.1097/01.CCM.0000239121.09533.09 16915117

[21] Vallés J Calbo E Anoro E Fontanals D Xercavins M Espejo E Bloodstream infections in adults: importance of healthcare-associated infections. J Infect Dis. (2008) 56(1):27–34. 10.1016/j.jinf.2007.10.001 18022242

[22] Berlak N Shany E Ben-Shimol S Chertok IA Goldinger G Greenberg D Late onset sepsis: comparison between coagulase-negative staphylococci and other bacteria in the neonatal intensive care unit. Infect Dis. (2018) 50(10):764–70. 10.1080/23744235.2018.1487075 29969049

[23] Marr I Swe K Henderson A Lacey JA Carter GP Ferguson JK. Cefazolin susceptibility of coagulase-negative staphylococci (CoNS) causing late-onset neonatal bacteraemia. J Antimicrob Chemother. (2022) 77(2):338–44. 10.1093/jac/dkab402 34791307

[24] May L Klein EY Rothman RE Laxminarayan R. Trends in antibiotic resistance in coagulase-negative staphylococci in the United States, 1999–2012. Antimicrob Agents Chemother. (2014) 58(3):1404–9. 10.1128/AAC.01908-13 24342646 PMC3957827

[25] Ericson JE Thaden J Cross HR Clark RH Fowler VG Benjamin DK No survival benefit with empirical vancomycin therapy for coagulase-negative staphylococcal bloodstream infections in infants. Pediatr Infect Dis J. (2015) 34(4):371. 10.1097/INF.0000000000000573 25760564 PMC4357312

[26] Amoah J Klein EY Chiotos K Cosgrove SE Tamma PD. Administration of a β-lactam prior to vancomycin as the first dose of antibiotic therapy improves survival in patients with bloodstream infections. Clin Infect Dis. (2022) 75(1):98–104. 10.1093/cid/ciab865 34606585

[27] Buetti N Atkinson A Kottanattu L Bielicki J Marschall J Kronenberg A Patterns and trends of pediatric bloodstream infections: a 7-year surveillance study. Eur J Clin Microbiol Infect Dis. (2017) 36(3):537–44. 10.1007/s10096-016-2830-6 27885442

[28] Thaden JT Ericson JE Cross H Bergin SP Messina JA Fowler VG Survival benefit of empirical therapy for staphylococcus aureus bloodstream infections in infants. Pediatr Infect Dis J. (2015) 34(11):1175–9. 10.1097/INF.0000000000000850 26222060 PMC4604046

[29] Cressman AM Macfadden DR Verma AA Razak F Daneman N. Empiric antibiotic treatment thresholds for serious bacterial infections: a scenario-based survey study. Clin Infect Dis. (2019) 69(6):930–7. 10.1093/cid/ciy1031 30535310

[30] Müller L Srinivasan A Abeles SR Rajagopal A Torriani FJ Aronoff-Spencer E. A risk-based clinical decision support system for patient-specific antimicrobial therapy (iBiogram): design and retrospective analysis. J Med Internet Res. (2021) 23(12):e23571. 10.2196/23571 34870601 PMC8686485

