# Supplementary material for: Estimating antibiotic coverage from linked microbiological and clinical data from the Swiss Paediatric Sepsis Study to support empiric antibiotic regimen selection
Source: Front Pediatr. 2023 May 11;11:1124165. doi: 10.3389/fped.2023.1124165 (PMC10213904; doi:10.3389/fped.2023.1124165)
Supplement: Supplementary file 1 [file Datasheet1.pdf]

Supplementary Material – Figures and Tables

Supplementary Figure 1.

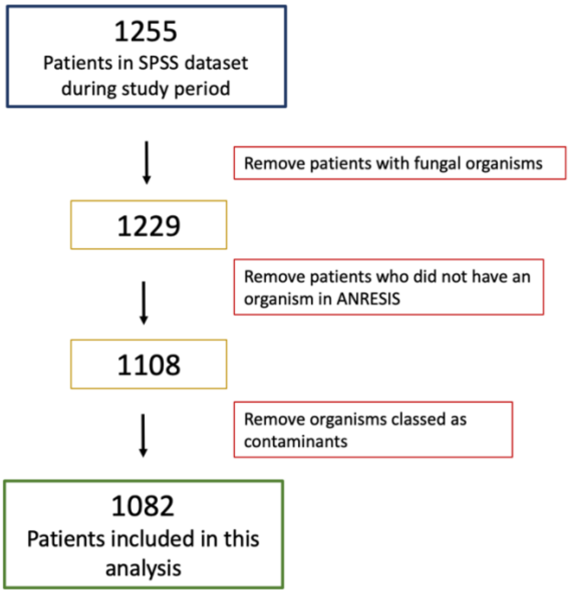

Supplementary Figure 1. Study schematic illustrating patients from SPSS included in this analysis after linkage to ANRESIS.

Supplementary Table 1

Supplementary Table 1. Number of patients in each risk group at each hospital.

	Patient group					
	**group1 - neonatal sepsis CAI**	**group2 - neonatal sepsis HAI**	**group3 - CAI healthy children**	**group4 - CAI comorbidity children**	**group5 - HAI children**	**Total**
Hospital						
Hospital 1	10 (18%)	13 (23%)	26 (46%)	7 (12%)	0 (0%)	56 (100%)
Hospital 2	11 (12%)	15 (17%)	34 (38%)	20 (22%)	9 (10%)	89 (100%)
Hospital 3	26 (16%)	18 (11%)	52 (32%)	44 (27%)	24 (15%)	164 (100%)
Hospital 4	7 (12%)	16 (28%)	24 (42%)	9 (16%)	1 (1.8%)	57 (100%)
Hospital 5	1 (1.3%)	29 (36%)	13 (16%)	15 (19%)	22 (28%)	80 (100%)
Hospital 6	22 (13%)	59 (34%)	33 (19%)	31 (18%)	29 (17%)	174 (100%)
Hospital 7	12 (14%)	12 (14%)	38 (46%)	12 (14%)	9 (11%)	83 (100%)
Hospital 8	7 (14%)	42 (86%)	0 (0%)	0 (0%)	0 (0%)	49 (100%)
Hospital 9	9 (8.5%)	20 (19%)	50 (47%)	16 (15%)	11 (10%)	106 (100%)
Hospital 10	17 (7.6%)	24 (11%)	58 (26%)	59 (26%)	66 (29%)	224 (100%)
**Total**	122 (11%)	248 (23%)	328 (30%)	213 (20%)	171 (16%)	1,082 (100%)

Supplementary Table 2

Supplementary Table 2. Overall summary of all pathogens isolated in the 10 study hospitals included in this analysis.

Pathogen	Number	Proportion
Escherichia coli	231	0.21
Coagulase-negative Staphylococcus	178	0.16
Staphylococcus aureus	172	0.16
Streptococcus pneumoniae	116	0.11
Viridans group Streptococcus	69	0.06
Klebsiella pneumoniae	42	0.04
Streptococcus agalactiae	46	0.04
Streptococcus pyogenes	41	0.04
Enterococcus faecalis	35	0.03
Enterobacter cloacae	26	0.02
Haemophilus spp.	18	0.02
Neisseria meningitidis	17	0.02
Citrobacter spp.	7	0.01
Enterococcus faecium	6	0.01
Klebsiella oxytoca	13	0.01
Pseudomonas aeruginosa	11	0.01
Salmonella spp.	9	0.01
Serratia spp.	6	0.01
Acinetobacter spp.	3	0.00
Campylobacter jejuni	1	0.00
Enterobacter aerogenes	2	0.00
Enterobacter spp.	3	0.00
Enterococcus species	5	0.00
Fusobacterium spp.	5	0.00
Gemella species	1	0.00
Granulicatella adiacens	1	0.00
Group C Streptococcus	2	0.00
Kingella kingae	1	0.00
Listeria monocytogenes	4	0.00
Moraxella catarrhalis	2	0.00
Morganella morganii	1	0.00
Neisseria spp.	5	0.00
Proteus mirabilis	2	0.00
Stenotrophomonas maltophilia	1	0.00

Supplementary Figure 2

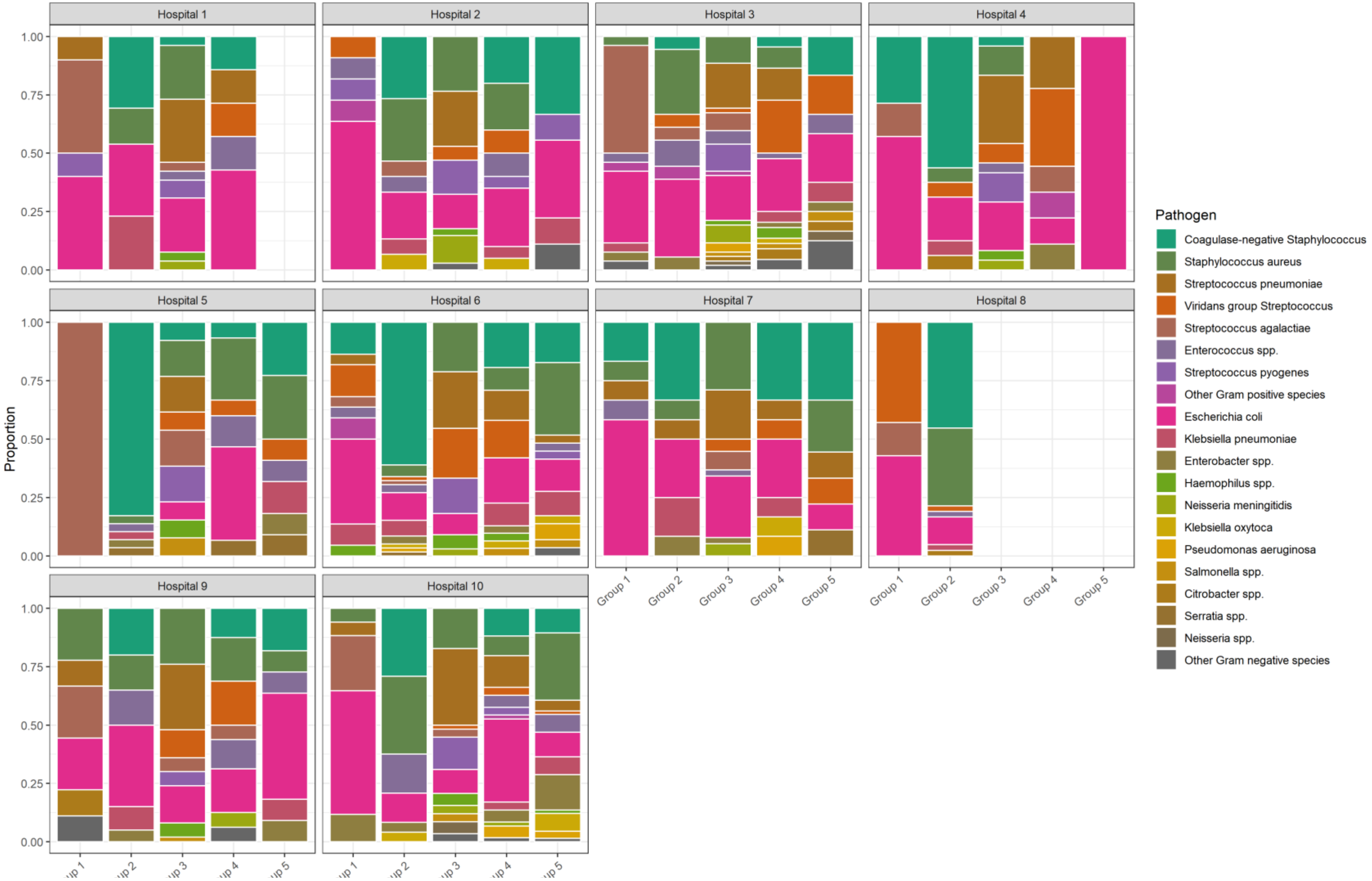

Supplementary Figure 2. Pathogen distribution by risk group at each hospital

Supplementary Figure 3.

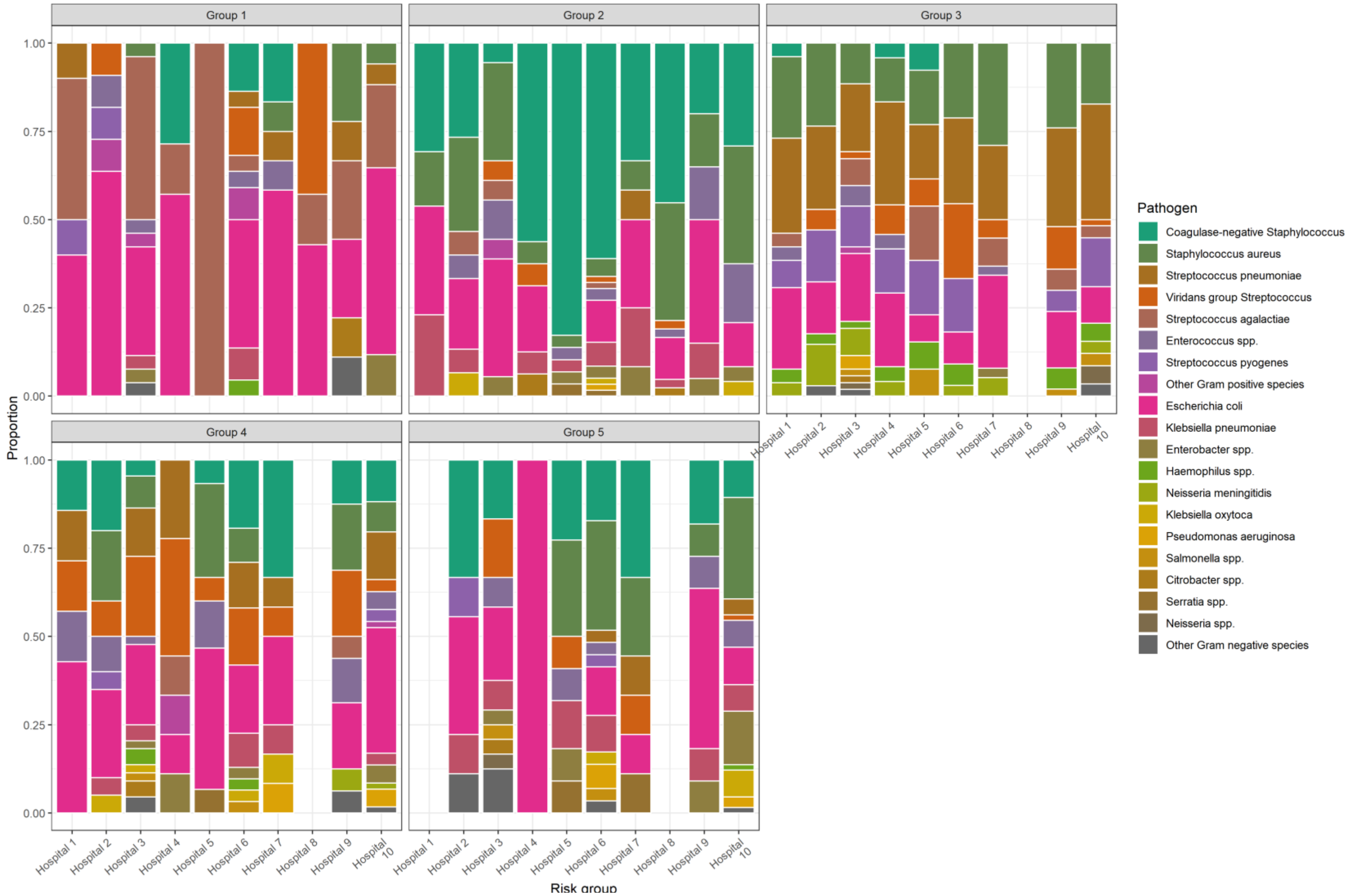

Supplementary Figure 3. Pathogen distribution by hospital for each risk group.

Supplementary Table 3

Supplementary Table 3. Coverage probability estimates and 95% credible intervals for each regimen of interest at each hospital.

Centre	Amoxicillin +Gentamicin	Amoxicillin/ clavulanic acid	Amoxicillin/ clavulanic acid +Gentamicin	Cefepime	Ceftriaxone	Ceftazidime +Amikacin	Piperacillin/ tazobactam	Meropenem
**Coverage pobability (95% CrI)**								
Hospital 1	0.91 (0.84-0.97)	0.82 (0.68-0.91)	0.88 (0.8-0.94)	0.82 (0.71-0.91)	0.81 (0.68-0.92)	0.56 (0.44-0.67)	0.86 (0.77-0.93)	0.86 (0.74-0.94)
Hospital 2	0.87 (0.79-0.93)	0.71 (0.62-0.79)	0.83 (0.75-0.9)	0.72 (0.62-0.8)	0.75 (0.66-0.84)	0.58 (0.48-0.68)	0.72 (0.62-0.81)	0.76 (0.65-0.85)
Hospital 3	0.81 (0.74-0.88)	0.68 (0.59-0.76)	0.83 (0.74-0.89)	0.74 (0.65-0.81)	0.76 (0.67-0.83)	0.49 (0.42-0.57)	0.76 (0.65-0.83)	0.83 (0.75-0.9)
Hospital 4	0.79 (0.69-0.88)	0.63 (0.51-0.74)	0.77 (0.67-0.86)	0.75 (0.64-0.84)	0.74 (0.63-0.83)	0.48 (0.37-0.59)	0.63 (0.5-0.76)	0.77 (0.66-0.86)
Hospital 5	0.57 (0.46-0.67)	0.41 (0.31-0.51)	0.57 (0.46-0.68)	0.52 (0.41-0.61)	0.51 (0.4-0.61)	0.46 (0.35-0.56)	0.54 (0.42-0.65)	0.53 (0.42-0.63)
Hospital 6	0.73 (0.67-0.79)	0.59 (0.53-0.66)	0.73 (0.66-0.78)	0.66 (0.59-0.73)	0.64 (0.57-0.7)	0.51 (0.44-0.58)	0.65 (0.58-0.72)	0.69 (0.62-0.76)
Hospital 7	0.86 (0.78-0.92)	0.67 (0.57-0.76)	0.84 (0.76-0.91)	0.76 (0.66-0.84)	0.76 (0.67-0.83)	0.64 (0.54-0.73)	0.72 (0.62-0.82)	0.79 (0.69-0.86)
Hospital 8	0.59 (0.48-0.71)	0.58 (0.45-0.7)	0.65 (0.52-0.76)	0.63 (0.51-0.75)	0.58 (0.45-0.7)	0.51 (0.38-0.63)	0.63 (0.49-0.75)	0.63 (0.51-0.75)
Hospital 9	0.87 (0.8-0.93)	0.77 (0.69-0.85)	0.88 (0.82-0.93)	0.78 (0.7-0.85)	0.78 (0.7-0.85)	0.51 (0.42-0.61)	0.8 (0.72-0.88)	0.82 (0.75-0.89)
Hospital 10	0.84 (0.79-0.89)	0.72 (0.67-0.78)	0.84 (0.79-0.88)	0.72 (0.63-0.79)	0.76 (0.7-0.81)	0.58 (0.51-0.64)	0.8 (0.75-0.85)	0.82 (0.76-0.86)

Supplementary Table 4.

Supplementary Table 4. Coverage probability estimates and 95% credible intervals for each regimen of interest for each patient risk group.

Risk Group	Amoxicillin +Gentamicin	Amoxicillin/ clavulanic acid	Amoxicillin/ clavulanic acid +Gentamicin	Cefepime	Ceftriaxone	Ceftazidime +Amikacin	Piperacillin/ tazobactam	Meropenem
				**Coverage pobability (95% CrI)**				
group1 - neonatal sepsis CAI	0.87 (0.81-0.92)	0.75 (0.68-0.82)	0.87 (0.81-0.92)	0.84 (0.77-0.9)	0.81 (0.74-0.87)	0.57 (0.48-0.65)	0.81 (0.74-0.87)	0.87 (0.81-0.93)
group2 - neonatal sepsis HAI	0.63 (0.57-0.69)	0.47 (0.41-0.53)	0.63 (0.57-0.69)	0.53 (0.47-0.59)	0.52 (0.46-0.57)	0.55 (0.49-0.61)	0.56 (0.5-0.63)	0.53 (0.47-0.59)
group3 - CAI healthy children	0.92 (0.89-0.95)	0.85 (0.81-0.89)	0.9 (0.86-0.93)	0.87 (0.83-0.91)	0.91 (0.88-0.94)	0.4 (0.34-0.45)	0.88 (0.84-0.92)	0.93 (0.88-0.96)
group4 - CAI comorbidity children	0.85 (0.8-0.89)	0.69 (0.63-0.75)	0.85 (0.8-0.9)	0.77 (0.71-0.82)	0.75 (0.69-0.81)	0.59 (0.53-0.66)	0.78 (0.72-0.84)	0.81 (0.75-0.86)
group5 - HAI children	0.78 (0.72-0.84)	0.56 (0.49-0.64)	0.8 (0.74-0.85)	0.62 (0.54-0.69)	0.62 (0.55-0.69)	0.68 (0.61-0.74)	0.69 (0.62-0.75)	0.74 (0.67-0.8)

Supplementary Table 5

Supplementary Table 5. Coverage probability estimates and 95% credible intervals for regimens of interest PLUS vancomycin at each hospital.

Centre	Amoxicillin +Gentamicin + Vancomycin	Amoxicillin/ clavulanic acid + Vancomycin	Amoxicillin/ clavulanic acid +Gentamicin + Vancomycin	Cefepime + Vancomycin	Ceftriaxone + Vancomycin	Ceftazidime +Amikacin + Vancomycin	Piperacillin/ tazobactam + Vancomycin	Meropenem + Vancomycin
				**Coverage pobability (95% CrI)**				
Hospital 1	0.91 (0.84-0.97)	0.85 (0.76-0.92)	0.88 (0.8-0.95)	0.88 (0.79-0.95)	0.88 (0.8-0.95)	0.88 (0.8-0.95)	0.87 (0.78-0.94)	0.92 (0.83-0.97)
Hospital 2	0.92 (0.86-0.97)	0.82 (0.75-0.88)	0.88 (0.81-0.94)	0.87 (0.79-0.92)	0.9 (0.84-0.95)	0.86 (0.79-0.92)	0.85 (0.78-0.91)	0.93 (0.87-0.97)
Hospital 3	0.9 (0.85-0.94)	0.73 (0.67-0.8)	0.87 (0.82-0.91)	0.85 (0.8-0.9)	0.86 (0.81-0.91)	0.85 (0.8-0.9)	0.85 (0.8-0.9)	0.95 (0.91-0.98)
Hospital 4	0.91 (0.84-0.96)	0.79 (0.69-0.88)	0.89 (0.8-0.95)	0.89 (0.81-0.95)	0.89 (0.81-0.95)	0.87 (0.79-0.94)	0.88 (0.79-0.94)	0.91 (0.83-0.96)
Hospital 5	0.88 (0.8-0.94)	0.76 (0.67-0.84)	0.87 (0.8-0.93)	0.91 (0.83-0.96)	0.9 (0.83-0.95)	0.91 (0.85-0.96)	0.91 (0.84-0.96)	0.92 (0.86-0.97)
Hospital 6	0.94 (0.9-0.97)	0.84 (0.79-0.89)	0.93 (0.9-0.96)	0.92 (0.88-0.96)	0.91 (0.86-0.95)	0.91 (0.86-0.94)	0.92 (0.88-0.95)	0.95 (0.92-0.98)
Hospital 7	0.93 (0.88-0.98)	0.79 (0.7-0.87)	0.91 (0.85-0.96)	0.91 (0.84-0.96)	0.9 (0.83-0.95)	0.91 (0.84-0.96)	0.88 (0.81-0.94)	0.93 (0.87-0.97)
Hospital 8	0.93 (0.85-0.98)	0.88 (0.78-0.95)	0.93 (0.85-0.98)	0.93 (0.86-0.98)	0.9 (0.81-0.96)	0.93 (0.85-0.98)	0.93 (0.85-0.98)	0.93 (0.85-0.98)
Hospital 9	0.93 (0.86-0.96)	0.82 (0.74-0.89)	0.9 (0.84-0.95)	0.87 (0.8-0.93)	0.88 (0.82-0.94)	0.87 (0.8-0.92)	0.87 (0.81-0.93)	0.92 (0.86-0.96)
Hospital 10	0.92 (0.89-0.95)	0.8 (0.75-0.85)	0.9 (0.86-0.93)	0.86 (0.76-0.92)	0.88 (0.84-0.92)	0.89 (0.85-0.93)	0.88 (0.84-0.92)	0.94 (0.9-0.97)

Supplementary Table 6

Supplementary Table 6. Coverage probability estimates and 95% credible intervals for regimens of interest PLUS vancomycin for each risk group.

Risk Group	Amoxicillin +Gentamicin + Vancomycin	Amoxicillin/ clavulanic acid + Vancomycin	Amoxicillin/ clavulanic acid +Gentamicin + Vancomycin	Cefepime + Vancomycin	Ceftriaxone + Vancomycin	Ceftazidime +Amikacin + Vancomycin	Piperacillin/ tazobactam + Vancomycin	Meropenem + Vancomycin
				**Coverage pobability (95% CrI)**				
group1 - neonatal sepsis CAI	0.91 (0.86-0.95)	0.8 (0.73-0.86)	0.91 (0.86-0.95)	0.9 (0.84-0.94)	0.88 (0.82-0.93)	0.9 (0.85-0.94)	0.87 (0.82-0.92)	0.93 (0.88-0.97)
group2 - neonatal sepsis HAI	0.97 (0.94-0.98)	0.86 (0.82-0.9)	0.96 (0.93-0.98)	0.96 (0.94-0.98)	0.95 (0.92-0.97)	0.96 (0.94-0.98)	0.94 (0.91-0.97)	0.97 (0.94-0.98)
group3 - CAI healthy children	0.95 (0.93-0.97)	0.87 (0.83-0.9)	0.91 (0.87-0.93)	0.9 (0.87-0.93)	0.95 (0.92-0.97)	0.87 (0.83-0.9)	0.91 (0.87-0.93)	0.96 (0.92-0.98)
group4 - CAI comorbidity children	0.93 (0.89-0.96)	0.8 (0.74-0.85)	0.91 (0.87-0.94)	0.91 (0.86-0.94)	0.89 (0.85-0.93)	0.91 (0.87-0.94)	0.91 (0.87-0.94)	0.94 (0.91-0.97)
group5 - HAI children	0.9 (0.86-0.94)	0.71 (0.64-0.77)	0.89 (0.85-0.93)	0.83 (0.76-0.89)	0.82 (0.77-0.88)	0.89 (0.85-0.93)	0.85 (0.79-0.9)	0.93 (0.89-0.97)
