# Supplementary material for: Estimating antibiotic coverage from linked microbiological and clinical data from the Swiss Paediatric Sepsis Study to support empiric antibiotic regimen selection
Source: Front Pediatr. 2023 May 11;11:1124165. doi: 10.3389/fped.2023.1124165 (PMC10213904; doi:10.3389/fped.2023.1124165)
Supplement: Supplementary file 2 [file Datasheet2.pdf]

Supplementary material – Linkage Methods

Background - linkage 2

Methods - linkage 2

Overall approach 2

Table S1: Bacterial species recorded from drop-down menu in SPSS database 2

Data cleaning and preparation 2

Probabilistic matching 3

Semi-deterministic matching 3

Linkage validation 3

Figure S1: Process for linking of Anresis and SPSS databases 4

Results - linkage 4

Table S2: Distribution of characteristics in SPSS and Anresis 4

Table S3: Data linkage quality for a subset of 213/1278 (16.7%) isolates from SPSS 6

Table S4: Matching errors by bacterial species for 1241 isolates in SPSS, semi-deterministic linkage 7

Table S5: Matching errors by bacterial species for 1241 isolates in SPSS, probabilistic linkage 8

References 9

Background - linkage

In many settings, surveillance databases contain large datasets on antimicrobial resistance, for example based on the collection of anonymous laboratory data from routine clinical care. For selection of suitable empiric antibiotic regimens, data from patients with the targeted infection syndrome needs to be clearly identified to avoid bias from inclusion of unrelated specimens. This could be facilitated by linkage to clinical data, if available, and would offer the opportunity of a more targeted interpretation of resistance data for clinical practice. In Switzerland, routine surveillance of antimicrobial resistance is based on electronic transfer of all microbiological data generated by participating laboratories in an anonymised format. Consequently, very limited information on source patients is available, and it can be challenging to establish which isolates come from patients experiencing a specific infection syndrome.

Methods - linkage

The optimal methods for data linkage are still debated. On the face of it, deterministic linkage of unique identifiers is the obvious choice. However, these may either not be available or reliable in the datasets to be linked, or there may be regulatory and legal constraints to their use. Probabilistic linkage approaches have therefore also been explored, but obviously have the potential for linkage errors and could introduce bias. We therefore used semi-deterministic and probabilistic linkage approaches and explored the quality of resulting data linkage between a neonatal and paediatric sepsis cohort and routine antimicrobial resistance surveillance before estimating antibiotic regimen coverage from the linked dataset.

Overall approach

We used a small set of variables expected to be widely available in any surveillance or quality management database of sepsis or bloodstream infection for linkage, focusing on site, age group, gender, causative species and day of blood culture. Information for causative species from the SPSS dataset was taken from both the drop-down menu selection for causative pathogen (options shown in table S1) and any information provided in a related free text field.

Table S1: Bacterial species recorded from drop-down menu in SPSS database

S. pneumoniae	E. coli
S. agalactiae (GBS)	Klebsiella spp.
S. pyogenes (GAS)	P. aeruginosa
Streptococcus, viridans group	N. meningitidis
Enterococcus spp.	H. influenzae
S. aureus	Other gram-negative
Coagulase-negative staphylococci	
Other gram-positive	

Data cleaning and preparation

Prior to linkage it is extremely important to first clean up the data sets to ease the linkage process (Sayer et al.). Information for causative species from the SPSS dataset was taken from both the drop-down menu selection for causative pathogen (options shown in table S1) and any information provided in a related free text field. Our data preparation process included, but was not limited to, the following activities:

- Remove punctuation, spaces, change all letters to lower case.
- Recoding different expression for missing (98 records such as "-", "n.a.", "nk", "no known", "not done").
- Tidying up overly long names or text fields (e.g. 12 records such as "2 bc pos, 2nd bc with e. faecium", "diplococci in blood culture, no growth") by extracting species names if available or treating as missing if species could not be identified.
- Excluding those pathogens only found in one or other of the data sets as linkage would be impossible (e.g. 110 pathogens were only found in ANRESIS such as "rhodococcus species", "pantoea species").

Probabilistic matching

For the probabilistic matching we use the "RecordsLinkage" package in R, blocking on site, gender, age, and year and month of the date the blood culture was taken. We did not require a perfect match on the day of blood culture, as minor discrepancies are expected between clinical record and laboratory record, e.g. minor deviations in terms of day culture was taken from patient and day culture arrived in the laboratory for blood cultures taken in the late evening. A standard string comparison was used to match the pathogen names in each of the data sets, based on the Jaro and Winkler algorithm. The function then automatically finds potential matches assigning each a probabilistic score (we used "epiWeights" as score function). By trial and error, we then defined a suitable score threshold as criteria for matching pairs.

Semi-deterministic matching

For the semi-deterministic approach we included some "fuzzy"-type matching rules for the pathogen field. Firstly, we exhaustively listed possible matches of the pathogens in both data sets before finding possible matches, possibly confounded by spelling (e.g. "bacillus" and "bacilus", "enterokokkus" and "enterococcus") and language differences since there was a mixture of English and German (e.g. "meningococcus" and "menigokokken"). We went on to add more intelligent definitions to identify specific groupings using the "%like%" notation in R/SQL (e.g. one group contained [fungal, candida, candidaalbicans, calbicans, candidaalbicans]; another ["groupastreptococci", "streptococcuspyogenes", "streptococcusalphahemolytic", "spyogenes", "alphahemolyticstreptococci", "alphastreptokokken", "groupastreptococcus" and "streptococcus", "gas"]).

The semi-deterministic method proceeded stepwise:
Step 1: Matching records on site, gender, age group, pathogen and day of blood culture. Those records not matched are passed to step 2.
Step 2: As above, but without site being allowing date of blood culture to be within a 5 day window of that for the SPSS data set. Again, those records not matched are passed to Step 3.
Step 3: As Step 1, but with a "fuzzy"-type match on pathogen leading to additional matched records.
Step 4: As Step 3, but also allowing blood cultures to be within a 5 day window.
Step 5: Matching records on gender, age within +1 group, fuzzy-type matching on pathogen and day of blood culture within a 5 day window.
Step 6: Matching only on site, blood culture within 5 days and fuzzy-type match on pathogen.
Step 7: Match only on site and fuzzy-type pathogen.

For each of the above steps, where multiple records were matched we chose the one with the nearest blood culture date, or failing this (albeit admittedly somewhat arbitrarily) the first record. Those records not matched in any of the steps were identified and manually reviewed. This latter set was also an interesting source of finding new potential matches for the fuzzy matching process defined previously.

Linkage validation

Validating the proposed matches against an appropriately defined gold standard is extremely important if we are to be able to benchmark our process and have confidence in our results (Harron et al., 2014, Harron et al., 2017) Linkage can introduce bias in terms of both false negatives (i.e. missing matches) and false positives (too optimistic matching) (e.g. Schmidlin et al., 2013, Lim et al., 2016).

We validated by comparing our methods with data for two sites where the records were matched by first finding actual patient identifiers in ANRESIS and SPSS on a case by case basis by hand, and then matching the appropriate records purely deterministically based on this unique key. This was done for 213/1278 (16.7%) of the SPSS cohort.

We then identified the overall number of unlinked records for the cohort and reviewed this by pathogen group as defined in SPSS. For each isolate we determined whether linkage had been achieved and whether the bacterial species had been correctly matched. This gives an overall indication of expected bias

in resistance estimates when resistance is determined from susceptibility testing from an incorrectly matched bacterial species with a potentially different expected resistance profile, for example enterococci and *Enterobacter* spp.

Figure S1: Process for linking of Anresis and SPSS databases

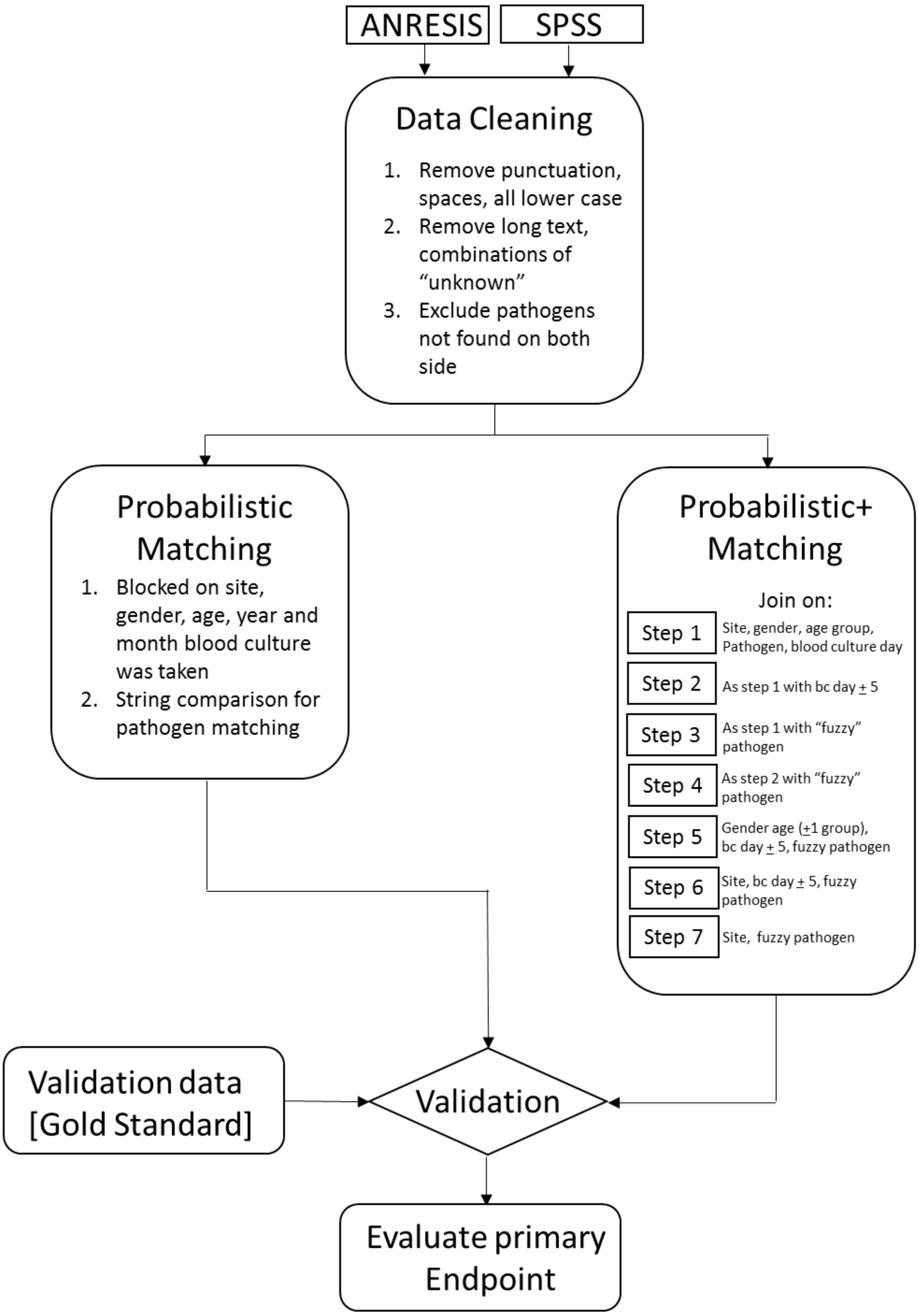

Results - linkage

In total, 1278 records were available in the final SPSS database. For the same period, 4268 pediatric records were contained in the Anresis database. The distribution of key characteristics in the two datasets is shown in Table S2.

Table S2: Distribution of characteristics in SPSS and Anresis

	SPSS (total 1278)	anresis (total 4268)

	N	%	N	%
**Site**				
Hospital 1	59	4.6	156	3.7
Hospital 2	102	8.0	432	10.1
Hospital 3	174	13.6	575	13.5
Hospital 4	70	5.5	85	2.0
Hospital 5	101	7.9	582	13.6
Hospital 6	211	16.5	420	9.8
Hospital 7	104	8.1	169	4.0
Hospital 9	136	10.6	26	0.6
Hospital 8 and Hospital 10	321	25.1	1270	29.8
Missing	0	0	553	13.0
**Gender**				
Female	518	40.5	1660	38.9
Male	760	59.5	2547	59.7
Missing	0	0	61	1.4
**Age group**				
≤ 2 yrs	774	60.6	2593	60.8
≤ 5 yrs	179	14.0	669	15.7
≤ 10 yrs	156	12.2	552	12.9
≤ 15 yrs	128	10.0	454	10.6
Missing	41	3.2	0	0
**Pathogen group**				
**Gram negative organisms**				
E. coli	242	18.9	392	9.2
Klebsiella spp.	55	4.3	118	2.8
N. meningitidis	28	2.2	21	0.5
P. aeruginosa	24	1.9	56	1.3
H. influenzae	22	1.7	22	0.5
Other Gram-negatives	82	6.4	282	6.6
**Gram positive organisms**				
Coagulase-negative staphylococci	184	14.4	1656	38.8
S. aureus	181	14.2	405	9.5
S. pneumoniae	119	9.3	276	6.5
S. agalactiae	105	8.2	101	2.4
Other streptococci	60	4.7	269	6.3
S. pyogenes	55	4.3	60	1.4
Enterococcus spp.	44	3.4	164	3.8
Other Gram-positive	40	3.1	269	6.3
**Fungal organisms**	23	1.8	137	3.2
**Missing/unknown**	14	1.1	40	0.9

The total number of SPSS records of interest for linkage excluding those lacking information on pathogen group and fungal isolates (not contributing to the estimation of antibiotic coverage) was 1241. Of these, 1119 (90%) were linked using the semi-deterministic approach (122/1241, 10% not linked) and 966 (78%)

were linked using the probabilistic approach (275/1241, 22% not linked). Linkage success in the gold standard data subset (n= 213, 16.7% of the total SPSS dataset) is shown in Table S3.

Table S3: Data linkage quality for a subset of 213/1278 (16.7%) isolates from SP*SS*

	**Semi-deterministic approach**		**Probabilistic approach**	
	N	%	N	%
**Hospital 3** total = 55				
Correctly matched	45	82	40	73
Incorrectly matched	0	0	3	5
Not matched	10	18	12	22
**Hospital 8 and Hospital 10** total = 158				
Correctly matched	135	85	118	75
Incorrectly matched	6	4	16	10
Not matched	17	11	24	15
**Overall** total = 213				
Correctly matched	180	84	158	74
Incorrectly matched	6	3	19	9
Not matched	27	13	36	17

For the semi-deterministic approach, linkage correctness by SPSS pathogen group was >90% for E. coli (95%), Klebsiella spp. (98%), coagulase-negative staphylococci (98%), Enterococcus spp. (98%), S. aureus (96%) and other streptococci (non-A, non-B, non-pneumococcal, 100%). Incorrect linkage based on non-matching bacterial species >10% was observed for N. meningitidis (18%), S. pneumoniae (15%) and S. pyogenes (29%). Linkage was missing for >10% of H. influenzae (14%), N. meningitidis (25%), P. aeruginosa (54%) and S. agalactiae (62%) (Table S4).

For the probabilistic approach, none of the SPSS pathogen groups achieved linkage correctness > 90% with the highest being 85% for *Klebsiella* spp and coagulase-negative staphylococci. Incorrect linkage >10% was observed only for *S. agalactiae* (19%). However, for all pathogen groups, linkage was not achieved in >10% of cases and was missing in >20% for *E. coli* (21%), *H. influenzae* (45%), *N. meningitidis* (25%), *Enterococcus* spp. (25%), *S. aureus* (25%), *S. pyogenes* (35%) and other streptococci (33%) (Table S5).

Table S4: Matching errors by bacterial species for 1241 isolates in SPSS, semi-deterministic linkage

	N in SPSS	Correct n(%)	Incorrect n(%)	Unmatched n(%)	Details of mismatches
E. coli	242	230 (95%)	5 (2%)	7 (3%)	*B. cereus* (1), *Citrobacter* spp. (1), *Enterobacter* spp. (2), *Enterococcus* spp. (1)
H. influenzae	22	17 (77%)	2 (9%)	3 (14%)	*H. parainfluenzae* (1); *H. aphrophilus* (1)
Klebsiella spp.	55	54 (98%)	1 (2%)	0	*Enterobacter* spp. (1)
N. meningitidis	28	16 (57%)	5 (18%)	7 (25%)	*N. cinerea* (1), *N. sicca* (1), *Neisseria* spp. (3)
P. aeruginosa	24	11 (46%)	0	13 (54%)	-
Coagulase-negative staphylococci (CoNS)	184	180 (98%)	3 (2%)	1 (1%)	*Candida* spp. (2), *Enterococcus* spp. (1)
Enterococcus spp.	44	43 (98%)	0	0	*B. cereus* (1)
S. aureus	181	174 (96%)	3 (2%)	4 (2%)	CoNS (1), *Enterococcus* spp. (1), *E. coli* (1)
S. agalactiae	105	33 (31%)	7 (7%)	65 (62%)	*Klebsiella* spp. (1), *S. pneumoniae* (4), *S. pyogenes* (1), *Streptococcus* spp. (1)
S. pneumoniae	119	101 (85%)	18 (15%)	0	*S. pyogenes* (2), *Streptococcus* spp. (16)
S. pyogenes	55	38 (69%)	16 (29%)	1 (2%)	*S. pneumoniae* (6), *Streptococcus* spp. (10)
Other streptococci	60	60 (100%)	0	0	

Other gram-positive bacteria=40, 9 unmatched, 29 matched with gram-positive bacteria, 3 matched with gram-negative bacteria, 0 matched with fungal isolates.
Other gram-negative bacteria=82, 12 unmatched, 7 matched with gram-positive bacteria, 62 matched with gram-negative bacteria, 1 matched with fungal isolates.

Table S5: Matching errors by bacterial species for 1241 isolates in SPSS, probabilistic linkage

	N in SPSS	Correct n(%)	Incorrect n(%)	Unmatched n(%)	Details of mismatches
E. coli	242	185 (76%)	5 (2%)	52 (21%)	CoNS. (3), *Enterobacter* spp. (1), *P. aeruginosa* (1)
H. influenzae	22	10 (45%)	2 (9%)	10 (45%)	*Enterococcus* spp. (1), *Granulicatella* spp.(1)
Klebsiella spp.	55	47 (85%)	1 (2%)	6 (11%)	*Enterobacter* spp. (1)
N. meningitidis	28	14 (50%)	7 (25%)	7 (25%)	*Staphylococcus* spp. (5), *S. agalactiae* (1), *S. pneumoniae* (1)
P. aeruginosa	24	19 (79%)	1 (4%)	4 (17%)	*Staphylococcus* spp. (1)
Coagulase-negative staphylococci (CoNS)	184	157 (85%)	4 (2%)	23 (13%)	*Candida* spp. (1), *Enterococcus* spp. (1), *Enterobacter* spp. (1), *E. coli* (1)
Enterococcus spp.	44	32 (73%)	1 (2%)	11 (25%)	*B. cereus* (1)
S. aureus	181	124 (69%)	11 (6%)	46 (25%)	Anaerobe (1), *Staphylococcus* spp. (9), *S. pneumoniae* (1)
S. agalactiae	105	64 (61%)	20 (19%)	21 (20%)	*Klebsiella* spp. (2), *S. pneumoniae* (2), *S. pyogenes* (1), *Streptococcus* spp. (2), *S. aureus* (1), *Staphylococcus* spp. (12)
S. pneumoniae	119	86 (72%)	10 (8%)	23 (19%)	*Fusobacterium* spp. (1), *P. aeruginosa* (1), *S. aureus* (2), *Staphylococcus* spp. (5), *Streptococcus* spp. (1)
S. pyogenes	55	33 (60%)	3 (5%)	19 (35%)	*S. pneumoniae* (1), *Staphylococcus* spp. (2)
Other streptococci	60	35 (58%)	5 (8%)	20 (33%)	*H. influenzae* (1), *Staphylococcus* spp. (4)

Other gram-positive bacteria=40, 13 unmatched, 26 matched with gram-positive bacteria, 1 matched with gram-negative bacteria, 0 matched with fungal isolates.

Other gram-negative bacteria =82, 20 unmatched, 6 matched with gram-positive bacteria, 56 matched with gram-negative bacteria, 0 matched with fungal isolates.

References

Sayers et al “Probabilistic record linkage”, Inter. J of Epi., 2016, pp 954-964

Harron K. et al “Evaluating bias due to data linkage error in electronic healthcare records”, BMC Med. Res. Meth 14(36), 2014

Harron K. et al. “Utilising identifier error variation in linkage of large administrative data sources”, BMC Med. Res. Meth 17(23), 2017

Schmidlin K. et al. “Impact of unlinked deaths and coding changes on mortality trends in the Swiss National Cohort”, BMC Medical Informatics 13(1), 2013.

Lim et al. “Optimization is required when using linked hospital and laboratory data to investigate respiratory infections”, J. Clin. Epi. 69, 2016, pp23-31.

R Core Team (2019). R: A language and environment for statistical computing. R Foundation for Statistical Computing, Vienna, Austria. URL https://www.R-project.org/.

M. Sariyar, A. Borg, “The RecordLinkage Package: Detecting Errors in Data”, The R Journal 2/2, December 2010.
